# The Clinical Features and Prognostic Assessment of SARS-CoV-2 Infection-Induced Sepsis Among COVID-19 Patients in Shenzhen, China

**DOI:** 10.3389/fmed.2020.570853

**Published:** 2020-10-15

**Authors:** Chao Ren, Ren-qi Yao, Di Ren, Jin-xiu Li, Ying Li, Xue-yan Liu, Lei Huang, Yong Liu, Mian Peng, Yao Yao, Yong-wen Feng, Yong-ming Yao

**Affiliations:** ^1^Department of Critical Care Medicine, The Second People's Hospital of Shenzhen, Shenzhen, China; ^2^Department of Critical Care Medicine, The Third People's Hospital of Shenzhen, Shenzhen, China; ^3^Trauma Research Center, Fourth Medical Center and Medical Innovation Research Division of the Chinese PLA General Hospital, Beijing, China; ^4^Department of Critical Care Medicine, The People's Hospital of Shenzhen, Shenzhen, China; ^5^Department of Critical Care Medicine, Peking University Shenzhen Hospital, Shenzhen, China; ^6^Department of Critical Care Medicine, Shenzhen Hospital of Southern Medical University, Shenzhen, China; ^7^Department of Critical Care Medicine, The Third Affiliated Hospital of Shenzhen University, Shenzhen, China; ^8^Center for Healthy Aging and Development Studies, National School of Development, Peking University, Beijing, China

**Keywords:** SARS-CoV-2, COVID-19, intensive care unit, sepsis, immune response

## Abstract

**Background:** The outbreak of the novel coronavirus disease (COVID-19) that began in December 2019 has posed a great threat to human health and caused a significant loss of life. In Shenzhen, 465 patients were confirmed to have COVID-19 as of August 31, 2020. In the present study, we aimed to describe the clinical characteristics of COVID-19 patients in Shenzhen and identify risk factors for the development of viral sepsis.

**Methods:** In this retrospective study, patients who were confirmed to have a severe acute respiratory syndrome coronavirus 2 (SARS-CoV-2) infection and were admitted to the Third People's Hospital of Shenzhen from January 11 to April 27, 2020 were included in the cohort. Clinical data were extracted and followed up to May 10, 2020, by using predesigned data collection forms.

**Results:** A total of 422 hospitalized COVID-19 patients were enrolled in this study, including 97 (23%) patients with viral sepsis at hospital admission and 325 (77%) non-septic patients. Patients with sepsis were much older than those without sepsis (57 vs. 43 years, *P* < 0.001) and presented with more comorbidities. Septic patients showed multiple organ dysfunction and significant abnormalities in immune- and inflammation-related biomarkers, and had poorer outcomes when compared to those without sepsis. Increased levels of interleukin-6, blood urea nitrogen, and creatine kinase were associated with the development of SARS-CoV-2-induced sepsis, and an elevated production of interleukin-6 was found to be an independent risk factor for the progression to critical illness among septic COVID-19 patients.

**Conclusions:** SARS-CoV-2 infection-induced sepsis is critically involved in the severity and prognosis of COVID-19 patients by characterizing both aberrant immune response and uncontrolled inflammation. The development of sepsis might contribute to multiple organ dysfunction and poor outcomes in COVID-19 patients during hospitalization.

## Introduction

Currently, the novel coronavirus disease (COVID-19) that was firstly reported in Wuhan, Hubei province, China, has caused significant loss of life (1, 2). Worldwide, as of August 31, 2020, a total of 24,854,140 patients have been diagnosed with COVID-19, and 838,924 cases resulted in death, which prompted the World Health Organization (WHO) to declare this a global pandemic (3, 4). From the first occurrence of COVID-19 on January 11, 2020, to August 31, 2020, 465 patients were confirmed with a severe acute respiratory syndrome coronavirus 2 (SARS-CoV-2) infection in Shenzhen, China, including one patient who remained in the hospital, three patients who died, and 462 patients who were discharged (5). However, data on the clinical features and outcomes of these COVID-19 cases and whether they developed septic complications remain scarce.

Sepsis is one of the leading causes of mortality in intensive care units (ICUs) and is characterized by multiple organ dysfunction due to an uncontrolled response to infection, according to the definition of sepsis 3.0 (6–8). Various pathogens, including bacteria, fungi, viruses, and even parasites, are deemed to induce sepsis by triggering aberrant immune responses in multiple organs, especially in the lungs (9). In fact, viral infection is commonly complicated by sepsis. A study by Zhou et al. (10) showed that ~40% of patients with influenza and non-influenza viral pneumonia developed sepsis during hospitalization, potentially accounting for higher ICU admissions and poorer outcomes. During the outbreak of severe acute respiratory syndrome (SARS), nosocomial sepsis was also a frequent complication in the ICU setting (11). Li and colleagues put forward a hypothesis on viral sepsis which stated that it might be crucial to the disease mechanism of COVID-19 (12). However, evidence in regard to the incidence of SARS-CoV-2-induced sepsis and associated clinical characteristics remains scarce in currently published reports on COVID-19.

## Methods

### Study Design and Participants

The current study was a single-center retrospective analysis conducted in the Third People's Hospital of Shenzhen, a tertiary care hospital in Shenzhen that was assigned by the local government to be a major center accounting for diagnosing and treating COVID-19 cases. Patients who were diagnosed with COVID-19 based on the World Health Organization interim guidance were all admitted to the Third People's Hospital of Shenzhen for isolation and treatment (13). All confirmed patients who were admitted to hospital from January 11 to April 27, 2020 were included in the cohort. The diagnosis of COVID-19 was in accordance with the World Health Organization interim guidance. Besides, clinical phenotypes of COVID-19 patients were classified as mild to critically ill cases in line with COVID-19 guidelines (the 7th edition) issued by the National Health Commission of the People's Republic of China. The criteria were presented as follows: mild cases: mild clinical symptoms with no sign of pneumonia on imaging; moderate cases: showing typical symptoms, including fever and respiratory symptoms with a radiological manifestation of pneumonia; severe cases: meeting one of the following criteria: (1) respiratory distress with respiratory rates ≥ 30 breaths/min; (2) oxygen saturation ≤ 93% on room air; (3) oxygenation index [arterial partial pressure of oxygen (PaO_2_)/fraction of inspired oxygen (FiO_2_)] ≤ 300; and critically ill cases: showing one of the following signs: (1) respiratory failure and requiring mechanical ventilation; (2) shock; (3) having other organ failure that requires ICU care. This study was approved by the committee on the ethics of medicine, the Second People's Hospital of Shenzhen, and oral consent was waived from each patient due to the urgent need for clinical data.

### Procedures

Specimens of the lower respiratory tracts, including bronchoalveolar lavage fluid (BALF), throat swabs, nasal swabs, and sputum of patients who were suspected of having COVID-19 were collected for SARS-CoV-2 RNA extraction. We used a QIAamp RNA Viral Kit (Qiagen, Heiden, Germany) to isolate viral RNA, which was subsequently tested through quantitative reverse transcription polymerase chain reaction (qRT-PCR) test. Both open reading frame 1ab (ORF1ab) and nucleocapsid protein (N) genes were targeted and measured by qRT-PCR assays using the China Food and Drug Administration (CFDA)-approved 2019-nCoV detection kit (GeneoDX Co., Ltd., Shanghai, China).

### Data Collection

Electronic medical records were used for data collection, and the clinical profile, nursing records, laboratory results, chest x-rays, and computed tomographic (CT) images were reviewed by the research team and the Expert Panel of Shenzhen COVID-19. Clinical data were extracted by using predesigned data collection forms. The disease onset was deemed from the date when the symptoms or signs were noticed by patients. Clinical outcomes including in-hospital death, discharge, and hospital and ICU lengths of stay were followed up to May 10, 2020. Plasma cytokine or chemokine levels were determined by applying a chemiluminescence method as well as the turbidimetric inhibition immunoassay. Absolute counts of peripheral blood T cells, CD4^+^ T cells and CD8^+^ T cells, and CD4^+^/CD8^+^ ratios were measured by flow cytometry. Clinical data, including laboratory findings and vital signs, were monitored on days 1, 3, 7, and 14 after hospital admission. Data from prognostic scoring systems, including the sequential organ failure assessment (SOFA) and acute physiology and chronic health evaluation II (APACHE II), were also obtained.

### Definition

The diagnosis of sepsis was based on the third international consensus definitions for sepsis and septic shock (Sepsis 3.0) criteria, which defined sepsis as SOFA score ≥ 2 plus documented or suspected infection (8). We solely considered sepsis caused by the SARS-CoV-2 infection, and patients with positive culture results at hospital admission were excluded from the sepsis cohort. Acute respiratory distress syndrome (ARDS) was diagnosed according to the Berlin criteria (14). The onset of acute kidney injury (AKI) was confirmed according to the kidney disease: improving global outcomes (KDIGO) definition (15). Patients whose conditions were complicated by cardiac injury were identified by means of cardiac biomarker serum levels higher than the 99th percentile upper reference limit or when new abnormalities were observed on electrocardiography and echocardiography (16). The diagnosis of coagulopathy was based on laboratory abnormalities in the coagulation profile.

### Statistical Analysis

The baseline characteristics of all enrolled patients in the sepsis and non-sepsis groups were summarized, and the groups were compared by applying Student's *t*-test, the Chi-square test, Fisher's exact test, and the Mann-Whitney *U* test as appropriate. Continuous variables were presented as the mean [standard deviation (SD)], standard error of the mean (SEM) or median [interquartile range (IQR)], while categorical or ranked data were reported as counts and proportions. Clinical indicators of repeated measures were compared between sepsis and non-sepsis groups by applying a general linear model. A two-tailed *P* < 0.05 was regarded as statistically significant.

We aimed to explore the risk factors related to the development of sepsis and factors in predicting critically ill cases among septic COVID-19 patients. Since numerous variables should be taken into account, the least absolute shrinkage and selection operator (LASSO) regression model was applied for variable selection correspondingly. The LASSO technique shrunk the coefficient estimated of unimportant variables toward zero by tuning parameter lambda. If it penalized some coefficient estimates to be exactly zero when the lambda was sufficiently large, instead, significant variables were retained. The most optimal lambda value was determined for which the cross-validation error was within one standard error of the minimum. Subsequently, selected variables combined with age and sex were included in the multivariate logistic regression model that adjusted for other significant risk factors. The odds ratio (OR) and 95% confidence interval (95% CI) were plotted accordingly.

The aforementioned statistical analyses were performed by using the Statistical Package for the Social Sciences (SPSS) software version 23.0 (SPSS Inc., Chicago, IL) as well as the R software version 3.6.1 (R Foundation for Statistical Computing, Vienna, Austria).

## Results

### Epidemiological Characteristics and Clinical Features

We included 422 hospitalized patients diagnosed with COVID-19 by April 27, 2020, and 99 (23.5%) were confirmed with septic complication based on increased SOFA scores. Finally, 97 (23.0%) cases were identified to have viral sepsis at hospital admission after excluding two patients with positive results in blood culture within 48 h after hospital admission. The median age of the COVID-19 patients in Shenzhen was 47 years (IQR, 33–60), and 221 (52.4%) patients were female (Table 1). The majority of patients [332 (78.7%)] had a history of travel to Hubei province, China. The most common comorbidities of these patients were hypertension [58 (13.7%)], followed by diabetes [22 (5.2%)], viral hepatitis type B [12 (2.8%)], coronary heart diseases [11 (2.6%)], and chronic bronchitis [6 (1.4%)]. The common symptoms were fever [290 (68.7%)], dry cough [126 (29.9%)], expectoration [81 [(19.2%)], fatigue [68 (16.1%)], and myalgia [59 (14.0%)]. Fifty-nine (14.0%) patients initially had no signs and symptoms. Clinical phenotypes of all the enrolled patients were classified into mild [264 (62.6%)], severe [109 (25.8%)], and critically ill [49 (11.6%)] cases. The median duration from the onset of symptoms to hospital admission was 3 days (IQR, 1–6), and the median length of stay in the hospital was 21 days (IQR, 16–29). A total of 376 (89.1%) patients were discharged from hospital, and 3 (0.7%) patients had died by April 27, 2020.

**Table 1:** Baseline characteristics of 422 patients confirmed with COVID-19 in Shenzhen, China.

	**No. (%) of patients**			
	**Total (*n* = 422)**	**Sepsis (*n* = 97)**	**Non-sepsis (*n* = 325)**	***P*-value**
**Demographic characteristics**				
Age, median (IQR), years	47.0 (33.0–60.0)	57.0 (41.0–64.0)	43.0 (33.0–57.0)	<0.001
Female	221 (52.4)	44 (45.4)	177 (54.5)	0.115
BMI, mean (SD)	23.1 (3.6)	23.6 (4.1)	23.0 (3.4)	0.23
Traveling history to Hubei province	332 (78.7)	73 (75.3)	259 (79.7)	0.349
**Comorbidities**				
Hypertension	58 (13.7)	25 (25.8)	33 (10.2)	<0.001
Diabetes	22 (5.2)	12 (12.4)	10 (3.1)	0.001
Viral hepatitis type B	12 (2.8)	3 (3.1)	9 (2.8)	>0.99
Coronary heart disease	11 (2.6)	3 (3.1)	8 (2.5)	>0.99
Chronic bronchitis	6 (1.4)	4 (4.1)	2 (0.6)	0.038
Smoking	6 (1.4)	2 (2.1)	4 (1.2)	0.906
Gout	5 (1.2)	1 (1.0)	4 (1.2)	>0.99
Malignant tumor	4 (0.9)	3 (3.1)	1 (0.3)	0.039
Cerebrovascular disease	2 (0.5)	2 (2.1)	0 (0)	0.052
Immunocompromised diseases	3 (0.7)	1 (1.0)	2 (0.6)	0.544
**Signs and symptoms**				
Fever	290 (68.7)	72 (74.2)	218 (67.1)	0.183
Dry cough	126 (29.9)	29 (29.9)	97 (29.8)	0.992
Expectoration	81 (19.2)	16 (16.5)	65 (20.0)	0.442
Fatigue	68 (16.1)	18 (18.6)	50 (15.4)	0.456
Myalgia	59 (14.0)	18 (18.6)	41 (12.6)	0.139
Chest distress	31 (7.3)	9 (9.3)	22 (6.8)	0.406
Dizziness	27 (6.4)	7 (7.2)	20 (6.2)	0.707
Headache	34 (5.5)	5 (5.2)	29 (8.9)	0.231
Anorexia	13 (3.1)	6 (6.2)	7 (2.2)	0.093
Diarrhea	23 (5.5)	5 (5.2)	18 (5.5)	0.884
Nausea	7 (1.7)	3 (3.1)	4 (1.2)	0.42
Dyspnea	8 (1.9)	2 (2.1)	6 (1.8)	>0.99
Stomach ache	5 (1.2)	1 (1.0)	4 (1.2)	>0.99
Vomiting	1 (0.2)	0 (0)	1 (0.3)	>0.99
No signs and symptoms	59 (14.0)	13 (13.4)	46 (14.2)	0.851
Body temperature, median (IQR), °C	37.0 (36.6–37.6)	37.4 (36.8–37.9)	36.9 (36.6–37.5)	<0.001
Heart rates, median (IQR), /min	88.0 (80.0–96.0)	90.5 (84.0–101.8)	86.0 (79.0–96.0)	0.002
Respiratory rates, median (IQR), /min	20.0 (19.0–20.0)	20.0 (20.0–22.0)	20.0 (19.0–20.0)	<0.001
MAP, median (IQR), mmHg	95.3 (89.0–103.7)	98.7 (92.3–107.0)	95.0 (88.3–103.0)	0.014
DBP, median (IQR), mmHg	81.0 (74.0–89.0)	82.0 (75.5–90.0)	81.0 (74.0–88.0)	0.272
SBP, median (IQR), mmHg	126.0 (116.0–137.3)	130.0 (120.5–144.0)	125.0 (115.0–136.0)	0.002
SOFA	1.0 (0.0–1.0)	2.0 (2.0–3.0)	1.0 (0.0–1.0)	<0.001
APACHE II	3.0 (2.0–6.0)	5.0 (3.0–7.0)	3.0 (1.0–5.0)	<0.001
Onset of symptoms to hospital admission, median (IQR), days	3.0 (1.0–6.0)	3.0 (2.0–6.0)	3.0 (1.0–5.5)	0.503
**Clinical phenotype^a^**				<0.001
Mild and moderate	264 (62.6)	41 (42.3)	223 (68.6)	
Severe	109 (25.8)	24 (24.7)	85 (26.2)	
Critical illness	49 (11.6)	32 (33.0)	17 (5.2)	
**Prognosis**				
Discharge from hospital	376 (89.1)	87 (89.7)	289 (88.9)	0.831
Length of stay in hospital, median (IQR), days	21.0 (16.0–29.0)	23.0 (17.5–34.5)	21.0 (16.0–28.0)	0.002
Hospital admission to ICU admission, median (IQR), mmHg	6.0 (2.0–9.0)	5.0 (2.0–8.0)	8.0 (6.0–10.0)	0.089
ICU admission	41 (9.7)	28 (28.9)	13 (4.0)	<0.001
Length of stay in ICU, median (IQR), days	14.0 (4.0–24.5)	14.0 (4.3–23.5)	16.0 (4.0–31.5)	0.866
In-hospital death	3 (0.7)	3 (3.1)	0 (0)	0.012

*IQR, interquartile range; SD, standard deviation; BMI, body mass index; MAP, mean arterial pressure; DBP, diastolic blood pressure; SBP, systolic blood pressure; SOFA, sequential organ failure assessment; APACHE II, acute physiology and chronic health evaluation II; ICU, intensive care unit*.

*Data were presented as median (IQR) or mean (SD). n (%) referred to the total number of patients with available data. P-values indicated differences between sepsis and non-sepsis patients, in which P < 0.05 was deemed as statistical significance*.

a *Clinical phenotype of COVID-19 patients was classified as mild to critically ill cases in line with COVID-19 guidelines (the 7th edition) issued by the National Health Commission of the People's Republic of China*.

Compared to patients without sepsis, those with SARS-CoV-2 infection-induced sepsis were significantly older [median age, 57 years (IQR, 41–64) vs. 43 years (IQR, 33–57); *P* < 0.001] and had more coexisting comorbidities, including hypertension [25 (25.8%) vs. 33 (10.2%); *P* < 0.001] and diabetes [12 (12.4%) vs. 10 (3.1%); *P* = 0.001]. Septic patients were more likely to be transferred to the ICU [28 (28.9%) vs. 13 (4.0%); *P* < 0.001] and had a significantly prolonged hospital stay [median days, 23 days (IQR, 17.5–34.5) vs. 21 days (IQR, 16–28); *P* = 0.002) than non-septic patients. Additionally, deaths [3 (3.1%)] occurred solely among patients who developed sepsis at hospital admission.

### Laboratory Findings

Septic patients had significantly higher alanine aminotransferase (ALT), aspartate aminotransferase, serum creatinine (sCr), blood urea nitrogen (BUN), creatine kinase (CK), lactate dehydrogenase, activated partial thromboplastin times, and D-dimer values than non-septic patients, but their monocyte counts, lymphocyte counts, platelet counts, and albumin levels were significantly lower (Supplementary Table 1). Significant differences in immune- or inflammation-related biomarkers were also noted between the sepsis group and the non-sepsis group and included decreased absolute counts and ratios of T lymphocytes and cytotoxic T lymphocytes as well as absolute helper T lymphocyte counts but increased levels of C-reactive proteins (CRP), and interleukin-6 (IL-6), and an increased erythrocyte sedimentation rate (ESR).

### Complications and Interventions

As shown in Table 2, 157 (37.2%) patients developed ARDS during hospitalization, and acute liver injury was diagnosed in 107 (25.4%) patients; other diagnoses included AKI in 19 (4.5%) patients, acute cardiac injury in 13 (3.1%) patients, shock in 9 (2.1%) patients, and coagulopathy in 11 (2.6%) patients. Notably, the incidence of multiple complications was significantly higher among patients with sepsis.

**Table 2:** Complications and treatments of patients with COVID-19 in Shenzhen, China.

	**No. (%) of patients**			
	**Total (*n* = 422)**	**Sepsis (*n*=97)**	**Non-sepsis (*n* = 325)**	***P-*value**
**Complications**				
ARDS	157 (37.2)	56 (57.7)	101 (31.1)	<0.001
Acute liver injury	107 (25.4)	42 (43.3)	65 (20.0)	<0.001
Acute kidney injury	19 (4.5)	16 (16.5)	3 (0.9)	<0.001
Acute cardiac injury	13 (3.1)	9 (9.3)	4 (1.2)	<0.001
Shock	9 (2.1)	7 (7.2)	2 (0.6)	<0.001
Coagulopathy	11 (2.6)	10 (10.3)	1 (0.3)	<0.001
**Treatments**				
Anti-virus therapy	422 (100.0)	97 (100.0)	325 (100.0)	NA
Interferon	372 (88.2)	89 (91.8)	283 (87.1)	0.211
Lopinavir and ritonavir tablets	350 (82.9)	83 (85.6)	267 (82.2)	0.433
Ribavirin	123 (29.1)	36 (37.1)	87 (26.8)	0.049
Oseltamivir	108 (25.6)	17 (17.5)	91 (28.0)	0.038
Favipiravir	67 (15.9)	15 (15.5)	52 (16.0)	0.391
Arbidol	125 (29.6)	27 (27.8)	98 (30.2)	0.661
Darunavir and cobicistat tablets	5 (1.2)	3 (3.1)	2 (0.6)	0.149
Acyclovir	4 (0.9)	3 (3.1)	1 (0.3)	0.039
Chinese traditional medicine	328 (77.7)	73 (75.3)	255 (78.5)	0.506
Human immunoglobulin	109 (25.8)	45 (46.4)	64 (19.7)	<0.001
Thymalfasin	175 (41.5)	58 (59.8)	117 (36.0)	<0.001
Glucocorticoid	108 (25.6)	43 (44.3)	65 (20.0)	<0.001
Antibiotics	152 (36.0)	45 (46.4)	107 (32.9)	0.015
NIV	47 (11.1)	30 (30.9)	17 (5.2)	<0.001
High-flow oxygen inhalation	18 (4.3)	10 (10.3)	8 (2.5)	0.002
IMV	16 (3.8)	12 (12.4)	4 (1.2)	<0.001
Chloroquine	30 (7.1)	5 (5.2)	25 (7.7)	0.393
CKRT	4 (0.9)	4 (4.1)	0 (0)	0.003
ECMO	2 (0.5)	2 (2.1)	0 (0)	0.052
Vasoactive agents	21 (5.0)	15 (15.5)	6 (1.8)	<0.001
Norepinephrine	16 (3.8)	10 (10.3)	6 (1.8)	<0.001
Adrenaline	17 (4.0)	11 (11.3)	6 (1.8)	<0.001
Dopamine	7 (1.7)	6 (6.2)	1 (0.3)	<0.001
Dobutamine	7 (1.7)	6 (6.2)	1 (0.3)	<0.001

*ARDS, acute respiratory distress syndrome; CKRT, continuous kidney replacement therapy; ECMO, extracorporeal membrane oxygenation; NIV, noninvasive ventilation; IMV, invasive mechanical ventilation*.

*Data were presented as n (%) that referred to the total number of patients with available data. P-values indicated differences between sepsis and non-sepsis patients, in which P < 0.05 was deemed as statistical significance*.

All patients had received anti-viral therapy during hospitalization, and the most frequently administrated drugs were interferon [372 (88.2%)], followed by lopinavir and ritonavir tablets [350 (82.9%)], ribavirin [123 (29.1%)], and oseltamivir [108 (25.6%)]. One hundred and fifty-two (36.0%) patients received antibiotics. Non-invasive ventilation (NIV), invasive mechanical ventilation (IMV), and high-flow oxygen inhalation were required in 47 (11.1%), 16 (3.8%), and 18 (4.3%) patients, respectively. Twenty-one (5.0%) patients received vasoactive agents. For those with organ support, 4 (0.9%) patients had continuous kidney replacement therapy (CKRT), and 2 (0.5%) patients received extracorporeal membrane oxygenation (ECMO) therapy.

### Dynamic Profile of Laboratory Findings

**Figure 1:**
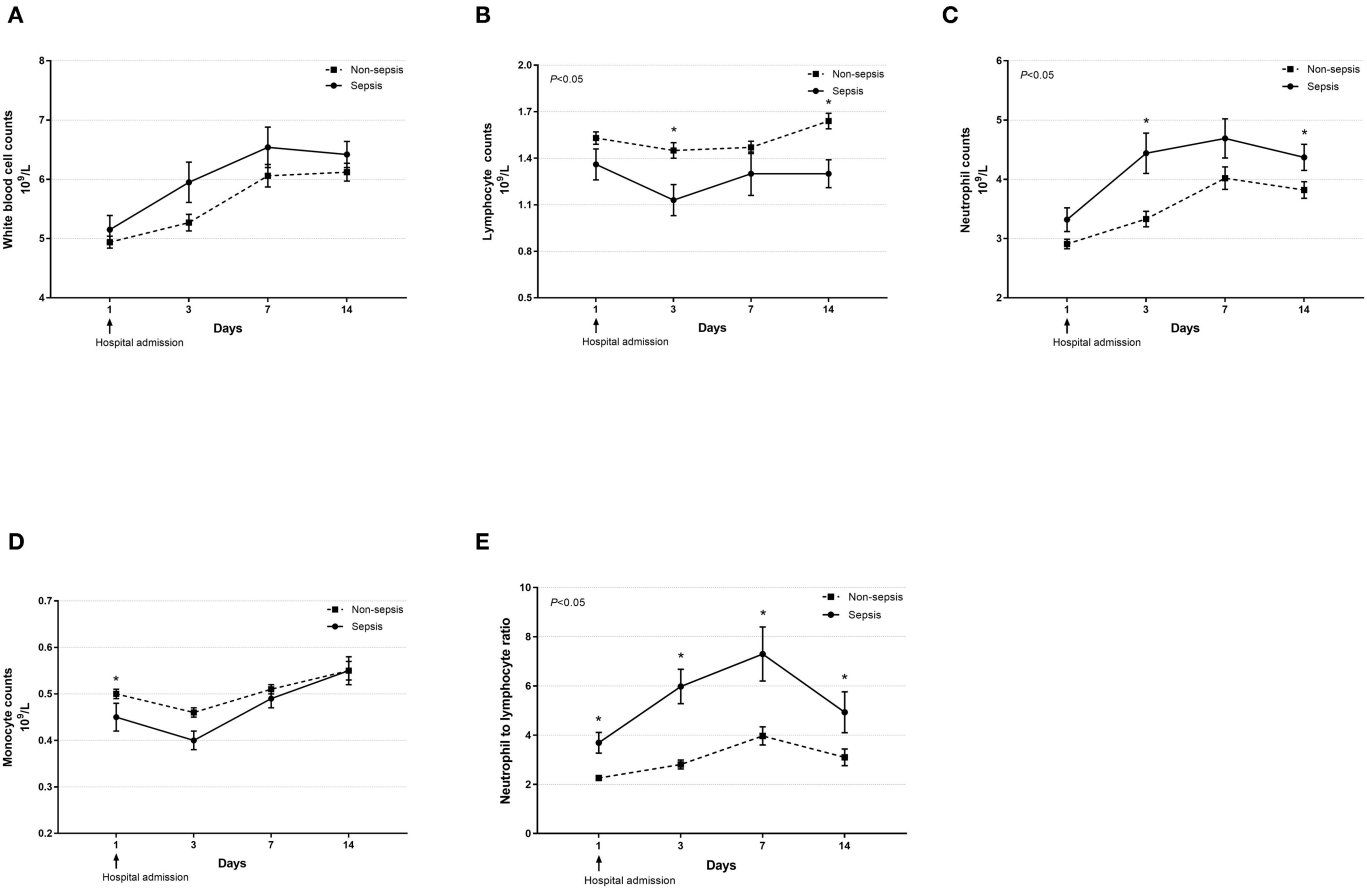
Dynamic profiles of cell counts in septic and non-septic COVID-19 patients. The counts of white blood cells **(A)**, lymphocytes **(B)**, neutrophils **(C)**, monocytes **(D)**, and neutrophil-to-lymphocyte ratio **(E)** in the peripheral blood obtained from septic and non-septic COVID-19 patients were monitored and compared on days 1, 3, 7, and 14 after hospital admission. Data were presented as mean ± SEM. ^*^P < 0.05, septic patients vs. non-septic patients. Results were tested for significance by using a general linear model with corrections for repeated measures.

**Figure 2:**
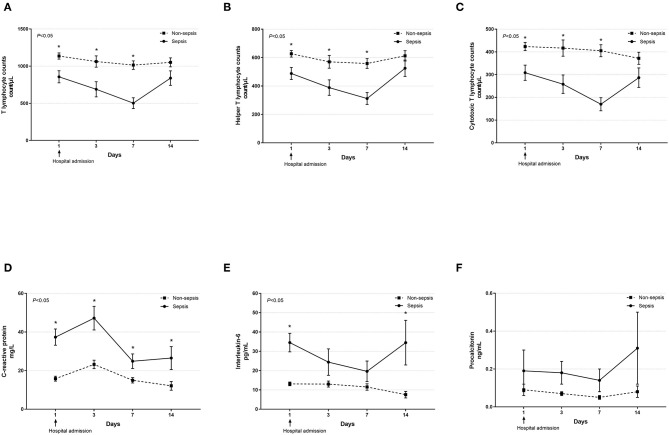
Dynamic profiles of cell counts of various T lymphocyte subsets and levels of inflammatory biomarkers in septic and non-septic COVID-19 patients. The absolute counts of T lymphocytes **(A)**, helper T lymphocytes **(B)**, and cytotoxic T lymphocytes **(C)** as well as serum levels of C-reactive protein **(D)**, interleukin-6 **(E)**, and procalcitonin **(F)** in septic and non-septic COVID-19 patients were monitored and compared on days 1, 3, 7, and 14 after hospital admission. Data were presented as mean ± SEM. *P < 0.05, septic patients vs. non-septic patients. Results were tested for significance by using a general linear model with corrections for repeated measures.

**Figure 3:**
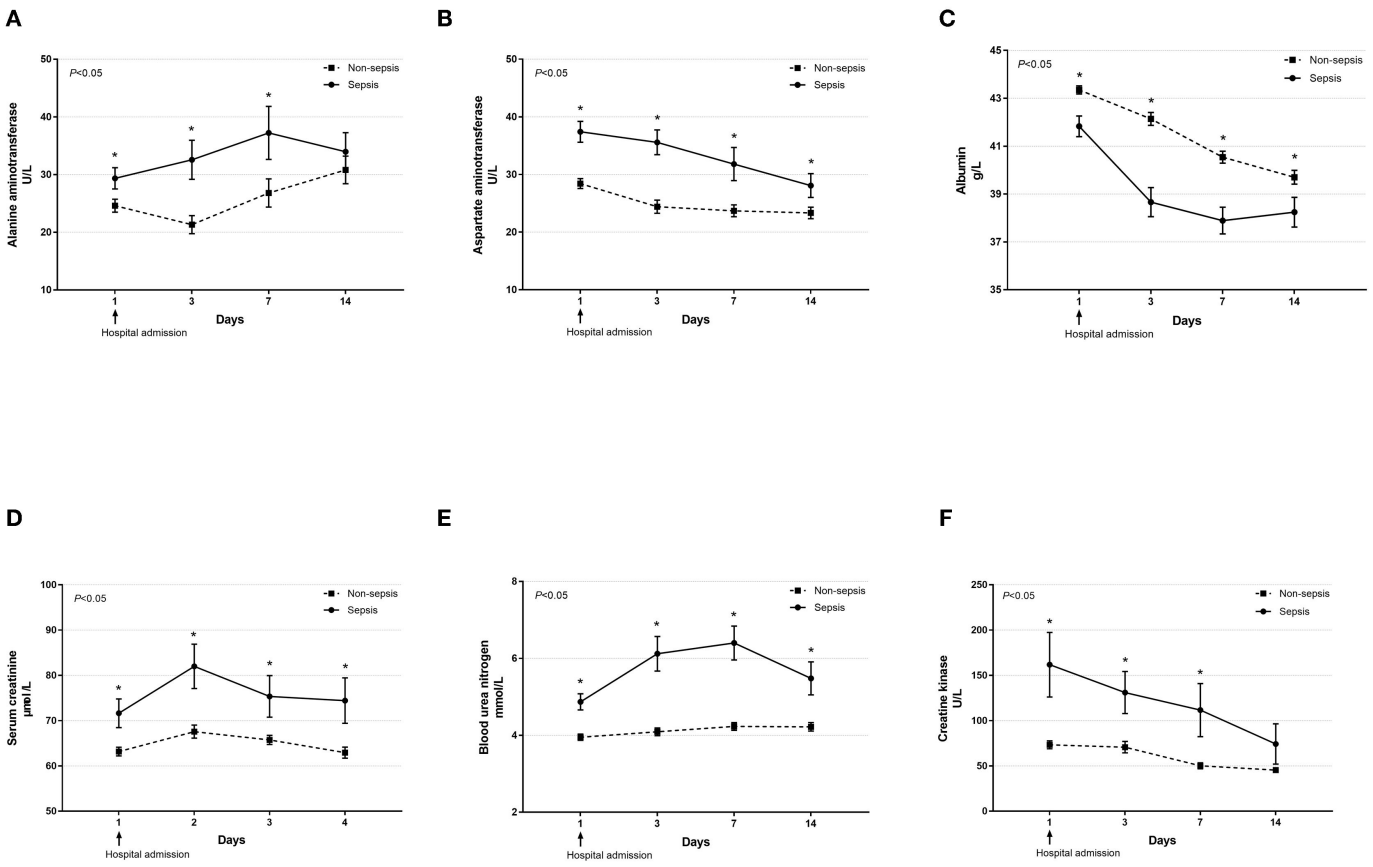
Dynamic profiles of indicators for organ function in septic and non-septic COVID-19 patients. The levels of alanine aminotransferase **(A)**, aspartate aminotransferase **(B)**, albumin **(C)**, serum creatinine **(D)**, blood urea nitrogen **(E)**, and creatinine kinase **(F)** in the peripheral blood obtained from septic and non-septic COVID-19 patients were monitored and compared on days 1, 3, 7, and 14 after hospital admission. Data were presented as mean ± SEM. *P < 0.05, septic patients vs. non-septic patients. Results were tested for significance by using a general linear model with corrections for repeated measures.

**Figure 4:**
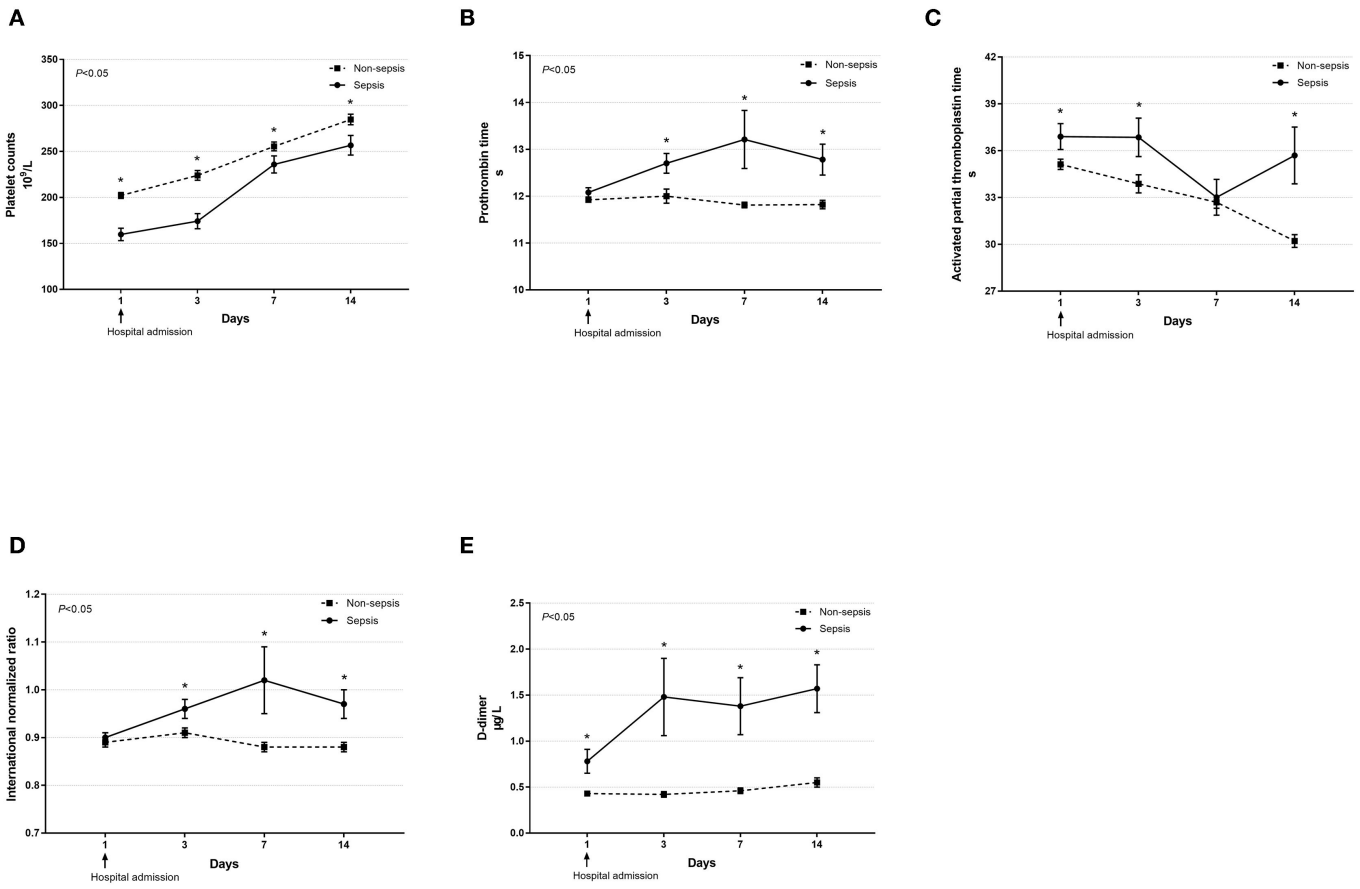
Dynamic profiles of indicators for coagulation function in septic and non-septic COVID-19 patients. Coagulation function, including platelet counts **(A)**, prothrombin time **(B)**, activated partial thromboplastin time **(C)**, international normalized ratio **(D)**, and D-dimer **(E)** in septic and non-septic COVID-19 patients were monitored and compared on days 1, 3, 7, and 14 after hospital admission. Data were presented as mean ± SEM. *P < 0.05, septic patients vs. non-septic patients. Results were tested for significance by using a general linear model with corrections for repeated measures.

Dynamic changes in cell counts, immune- and inflammation-related indicators, parameters for organ function as well as coagulation markers were recorded on days 1, 3, 7, and 14 after admission for hospitalized COVID-19 patients. As presented in Figure 1, most septic patients had lymphopenia and an elevated neutrophil-to-lymphocyte ratio (NLR) during the first three hospitalized days. Absolute counts of T lymphocytes, helper T lymphocytes, and cytotoxic T lymphocytes were significantly decreased in patients with sepsis and reached the lowest value on day 7, while levels of CRP and IL-6 were significantly higher at hospital admission but continually decreased after day 3 (Figure 2). Also, general linear model analysis revealed statistical significance between the two groups for all tracked organ function related parameters, in which septic patients had remarkably altered levels of ALT, ALB, sCr, and BUN on days 3 and 7 since hospital admission (Figure 3). As for coagulation indictors, septic COVID-19 patients had significantly altered coagulation compared to patients without sepsis (Figure 4).

### Risk Factors for SARS-CoV-2 Infection-Induced Sepsis

**Figure 5:**
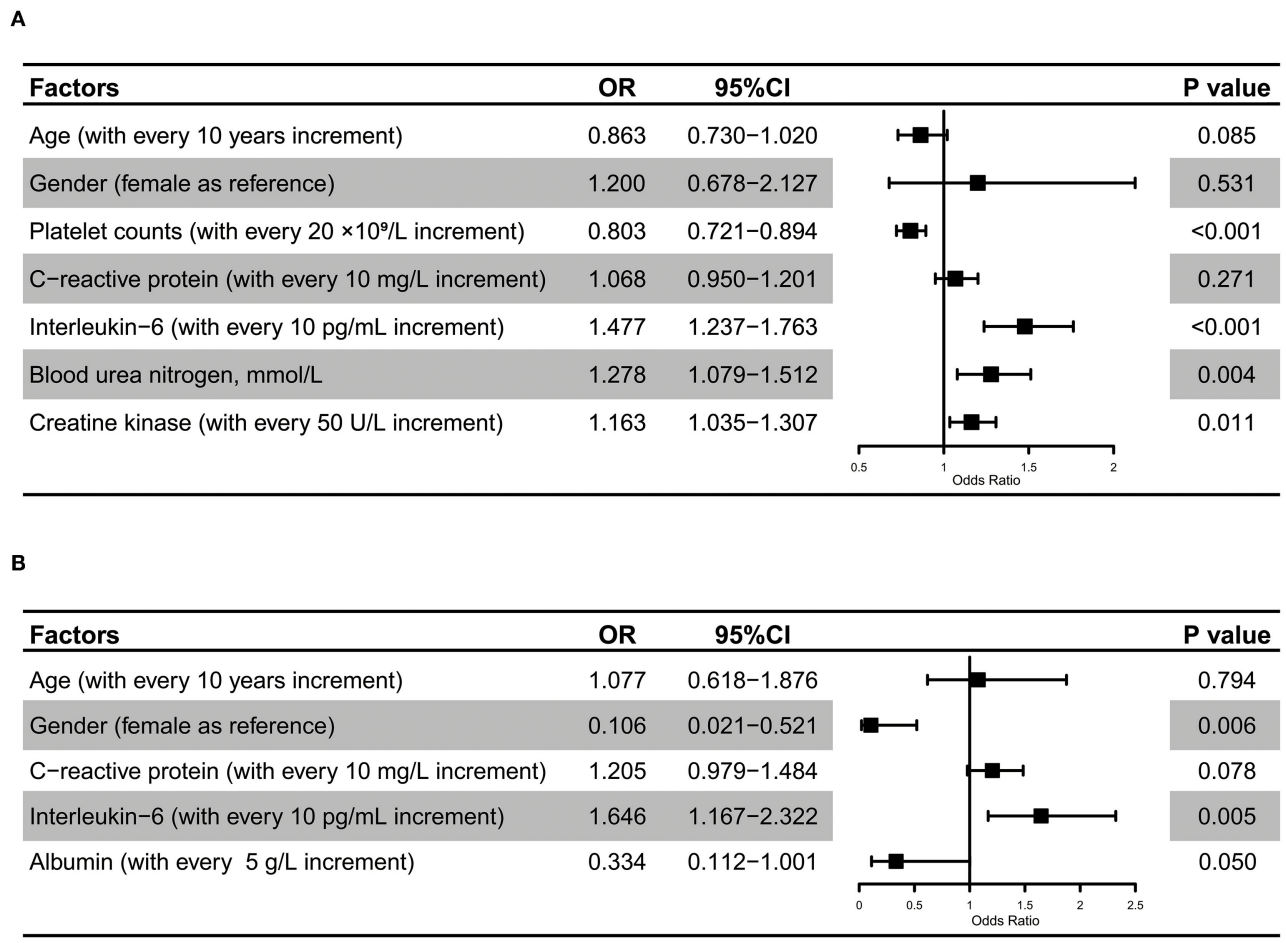
Risk factors for SARS-CoV-2 infection-induced sepsis. Multivariate regression analysis revealed that IL-6, BUN, and CK levels, and platelet counts were significantly associated with the development of SARS-CoV-2 infection-induced sepsis **(A)**. Multivariate regression analysis revealed that gender and IL-6 were significantly associated with the progression to critically ill cases among septic COVID-19 patients **(B)**.

By using LASSO regression analysis, we further identified risk factors for the development of sepsis (Figure 5, Supplementary Figure S1) and found that changes in platelet counts, CRP, IL-6, BUN, and CK levels might be useful for predicting the incidence of viral sepsis among COVID-19 cases. After adjustments for covariates, including age and sex, we found that a higher IL-6 level (OR for each 10 pg/mL increase, 1.477; 95% CI, 1.237–1.763; *P* < 0.001), increased BUN level (OR for each 1 mmol/L increase, 1.278; 95% CI, 1.079–1.512; *P* = 0.004), and higher CK level (OR for each 50 U/L increase, 1.163; 95% CI, 1.035–1.307; *P* = 0.011) were associated with the development of SARS-CoV-2 infection-induced sepsis, while a greater value of platelet count (OR for each 20 × 10^9^/L increase, 0.803; 95% CI, 0.721–0.894; *P* < 0.001) was associated with the decreased probability of developing sepsis. In addition, an elevated IL-6 level (OR for each 10 pg/mL increase, 1.646; 95% CI, 1.167–2.322; *P* = 0.005) was noticed to be independently associated with the progression to critically ill cases among septic COVID-19 patients.

## Discussion

In this study, we reported a cohort of 422 patients with a laboratory confirmed SARS-CoV-2 infection in Shenzhen, China. The demographic characteristics of these cases were quite similar to those in both Wuhan and other areas outside Hubei province, China (16, 17). For example, like reports in Zhejiang and Shanghai (17, 18), these imported cases presented with mild symptoms, as only 1.9% of patients developed dyspnea and 14% of patients were confirmed to have COVID-19 without the associated signs or symptoms. The majority of cases were recorded as mild based on COVID-19 guidelines (the 7th edition), and presented with relatively few complications. As of April 27, 2020, 9.7% of patients received ICU care, 89.1% were discharged from the hospital, and 3 (0.7%) died, indicating a lower mortality rate than that reported in Wuhan (19, 20).

We further evaluated the incidence of SARS-CoV-2 infection-induced sepsis at hospital admission, which was noteworthy in COVID-19 patients due not only to its lethal status but also to its etiology regarding the triggering pathogen, abnormal host immune response, and multiple organ dysfunction. In this study, a total of 97 (23%) COVID-19 patients were confirmed to have viral sepsis after excluding patients with additional infection at hospital admission. Patients with sepsis were much older and showed significant differences in indicators of organ dysfunction compared with non-septic patients, which might bring about more ICU admissions, prolonged hospital stays, and deaths. Of note, some indicators that showed significant differences between septic and non-septic patients had only limited absolute differences, which might be detected within the compensatory stage, and thus, the data should be interpreted cautiously based on distinct clinical practices. In fact, sepsis has been extensively discussed during the outbreak of other viral infections, such as SARS, influenza A virus, and Middle East respiratory syndrome coronavirus (MERS-CoV) (10, 11, 21). Generally, viruses are the causative organisms responsible for the development of sepsis due to their strong ability to disrupt the immune response and induce uncontrolled inflammation (22). Reports by Zhou and colleagues analyzed 951 patients with viral infections and revealed that only 6.9% of these patients were coinfected with viruses and bacteria, but the incidence of sepsis reached 40.1%, which provided direct evidence of viral sepsis (10). Nevertheless, the prevalence and pathophysiology of viral sepsis remain unclarified (23). Viral sepsis is prone to being neglected and is usually reported as nosocomial infection syndrome. For example, sepsis is considered a frequent complication in patients with SARS based on positive blood cultures and nosocomial pneumonia (11). Even though there are many difficulties in determining whether sepsis is initiated or acquired by viral infection, there is no doubt that the development of sepsis or septic shock results in poor outcomes (21).

The dissonance of the immune response to viral infections accounts for the major pathophysiology of sepsis. In the present study, there was a significant reduction in T lymphocytes in patients with sepsis compared to patients without sepsis, and this reduction was a difficult issue to resolve and was even deemed to be a unique characteristic of severe viral infection (24, 25). However, neither neutrophils nor monocytes showed an obvious increase during the hospital stay, and the NLR was significantly higher in septic patients than in non-septic patients, suggesting that an imbalanced response between the innate and adaptive immune systems might become a vicious cycle for the organ dysfunction. The presence of T lymphocytes is necessary for efficient clearance of local pathogens and is also of great importance in restricting the overactivated innate immune response due to its strong immunomodulatory capacity and critical involvement in neuroendocrine-immune networks (26, 27). The loss of T lymphocytes has been demonstrated to promote an excessive response by local innate immune cells. In return, overactivated innate immune cells, such as alveolar macrophages, are capable of inhibiting T cell priming and promote apoptosis by interfering with the activation of dendritic cells during viral infections (28). Thus, efficient prediction and timely interference in this vicious cycle might be an effective remedy for COVID-19 patients with sepsis.

The hyper-inflammatory response is regarded as an important cause of lung injury in COVID-19 patients (29, 30). Herein, CRP and IL-6 levels were significantly higher in patients with sepsis than in those without sepsis at hospital admission. Furthermore, increased levels of IL-6, BUN, and CK were associated with the development of SARS-CoV-2 infection-induced sepsis, indicating that an uncontrolled inflammatory response and multiple organ dysfunction were major characteristics for viral sepsis. The increase in IL-6 production was found to be an independent risk factor for the progression to critical illness among septic COVID-19 patients, which was consistent with the report by Wang and colleagues (29). A cytokine storm is deemed to be the main cause but is a difficult issue to resolve for multiple organ damage induced by severe viral infection; moreover, the source of cytokines varies and remains vague. The dysregulated immune response appears to be one of the major contributors for the high levels of cytokines due to hyperactivation of both resident and infiltrated inflammatory cells (30). Therefore, both immunomodulation and anti-inflammation might be effective remedies for improving the prognosis of COVID-19 patients. From the current data, 41.5% and 25.6% of septic patients received thymalfasin and glucocorticoids, respectively, on the basis of routine anti-viral therapies. It is worth noting that 77.7% of septic patients underwent treatment with Chinese traditional medicine, such as Xuebijing injection, partly due to its effects in terms of both immunomodulation and anti-inflammation (31, 32).

Several limitations should be noted when interpreting our findings. Firstly, even though a cohort of 422 COVID-19 patients was included in our analysis, a larger sample size, especially from multiple clinical centers outside Wuhan, China, is necessary to obtain more detailed information and determine the distinct clinical features of imported COVID-19 patients. Secondly, with the limited number of cases, it is difficult to assess the risk factors and further establish a predictive model for the occurrence of sepsis in patients with COVID-19. Thirdly, the development of nosocomial sepsis, which is recognized as a common complication of severe viral infection, also needs further investigation.

## Conclusions

In summary, viral sepsis is critically involved in the severity and prognosis of patients with a SARS-CoV-2 infection by characterizing both an aberrant immune response and uncontrolled inflammation. The development of sepsis might be responsible for, at least in part, multiple organ injury and poor outcomes in COVID-19 patients during hospitalization.

## Data Availability Statement

The raw data supporting the conclusions of this article will be made available by the authors, without undue reservation.

## Ethics Statement

The studies involving human participants were reviewed and approved by the committee on the ethics of medicine, the Second People's Hospital of Shenzhen. Written informed consent for participation was not required for this study in accordance with the national legislation and the institutional requirements.

## Author Contributions

Y-wF, Y-mY, CR, R-qY, and DR contributed to study concept and design. DR extracted epidemiological and clinical data. CR and R-qY performed the statistical analyses. J-xL, YLi, X-yL, LH, YLiu, and MP recruited patients. CR, R-qY, and DR co-drafted the initial version of the manuscript. YY was consulted for statistical and technical issues. Y-wF was responsible for the integrity and accuracy of the data and was the guarantor. Y-wF and Y-mY attested that all listed authors meet authorship criteria and that no others meeting the criteria have been omitted. All authors provided critical revision of the manuscript and approved the final draft for publication.

## Conflict of Interest

The authors declare that the research was conducted in the absence of any commercial or financial relationships that could be construed as a potential conflict of interest.

## Supplementary Material

Supplementary Figure S1 Variable selection process via the LASSO regression model. LASSO regression analysis coefficients for the risk factors of the onset of SARS-CoV-2 infection-induced sepsis **(A)**. LASSO regression analysis coefficients for the factors associated with the progression to critically ill cases among septic COVID-19 patients **(B)**. Selection of tuning parameters in the LASSO regression analysis based on 10 cross-validations in regard to the onset of SARS-CoV-2 infection-induced sepsis **(C)**, and the progression to critically ill cases among septic COVID-19 patients **(D)**.
