# Supplementary figures and images for: The Clinical Features and Prognostic Assessment of SARS-CoV-2 Infection-Induced Sepsis Among COVID-19 Patients in Shenzhen, China
Source: Front Med (Lausanne). 2020 Oct 15;7:570853. doi: 10.3389/fmed.2020.570853 (PMC7593782; doi:10.3389/fmed.2020.570853)

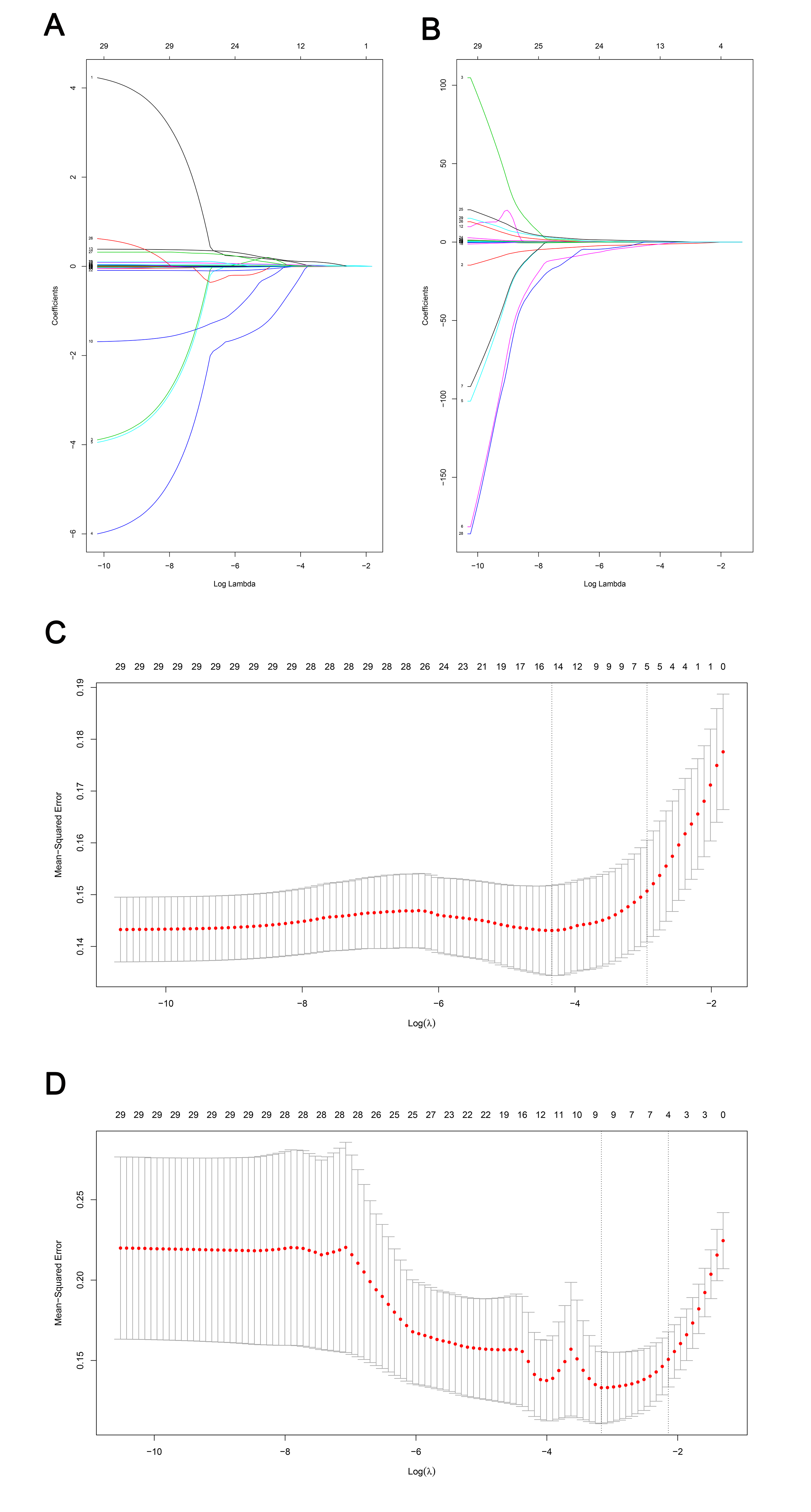

Supplement: Supplementary Figure S1 — Variable selection process via the LASSO regression model. LASSO regression analysis coefficients for the risk factors of the onset of SARS-CoV-2 infection-induced sepsis (A). LASSO regression analysis coefficients for the factors associated with the progression to critically ill cases among septic COVID-19 patients (B). Selection of tuning parameters in the LASSO regression analysis based on 10 cross-validations in regard to the onset of SARS-CoV-2 infection-induced sepsis (C), and the progression to critically ill cases among septic COVID-19 patients (D). [file Image_1.tif]
