# Supplementary material for: The Clinical Features and Prognostic Assessment of SARS-CoV-2 Infection-Induced Sepsis Among COVID-19 Patients in Shenzhen, China
Source: Front Med (Lausanne). 2020 Oct 15;7:570853. doi: 10.3389/fmed.2020.570853 (PMC7593782; doi:10.3389/fmed.2020.570853)
Supplement: Supplementary file 2 [file Table_1.docx]

**Supplemental Table S1. Comparisons of laboratory findings between septic and non-septic COVID-19 patients in Shenzhen, China**

|  |  | No. (%) of patients | | | |
| --- | --- | --- | --- | --- | --- |
|  | Normal range | Total (n=422) | Sepsis (n=97) | Non-sepsis (n=325) | *P* value |
| **Blood routine test** | | | | | |
| White blood cell counts, ×10^9^/L | 3.5-9.5 | 4.6 (3.8-5.8) | 4.6 (3.7-6.2) | 4.6 (3.8-5.7) | 0.92 |
| Neutrophil counts, ×10^9^/L | 1.8-6.3 | 2.7 (2.0-3.6) | 2.7 (2.0-4.5) | 2.7 (2.0-3.5) | 0.271 |
| Lymphocyte counts, ×10^9^/L | 1.1-3.2 | 1.3 (1.0-1.9) | 1.1 (0.8-1.6) | 1.4 (1.1-1.9) | <0.001 |
| Neutrophil to lymphocyte ratio | NA | 1.9 (1.3-3.0) | 2.4 (1.4-4.4) | 1.8 (1.3-2.8) | 0.002 |
| Monocyte counts, ×10^9^/L | 0.1-0.6 | 0.5 (0.3-0.6) | 0.4 (0.3-0.6) | 0.5 (0.4-0.6) | 0.004 |
| Platelet counts, ×10^9^/L | 125-350 | 186.0 (148.0-227.0) | 139.5 (117.8-184.5) | 193.0 (161.0-234.0) | <0.001 |
| Haemoglobin, mean (SD), g/L | 130-175 | 136.5 (126.0-146.0) | 136.0 (124.0-148.8) | 137.0 (127.0-146.0) | 0.579 |
| Hematocrit, mean (SD), % | 40.0-50.0 | 40.1 (37.3-43.3) | 39.2 (36.8-43.2) | 40.4 (37.6-43.4) | 0.156 |
| **Coagulation function** | | | | | |
| Prothrombin time, s | 10.5-13.5 | 12.0 (11.3-12.6) | 12.1 (11.5-12.7) | 11.9 (11.3-12.5) | 0.099 |
| Activated partial thromboplastin time, s | 21.0-37.0 | 35.1 (32.0-38.3) | 36.2 (33.7-39.0) | 34.7 (31.8-37.9) | 0.015 |
| International normalized ratio | 0.8-1.3 | 0.9 (0.8-1.0) | 0.9 (0.8-1.0) | 0.9 (0.8-0.9) | 0.104 |
| D-dimer, μg/L | 0.0-1.5 | 0.4 (0.3-0.5) | 0.4 (0.3-0.8) | 0.4 (0.3-0.5) | <0.001 |
| **Blood biochemistry** | | | | | |
| Albumin, mean (SD), g/L | 40.0-55.0 | 43.0 (41.0-45.2) | 42.2 (38.5-45.1) | 43.1 (41.3-45.3) | 0.004 |
| Alanine aminotransferase, U/L | 9.0-50.0 | 21.0 (15.0-31.2) | 24.9 (16.0-36.9) | 20.0 (14.8-29.0) | 0.009 |
| Aspartate aminotransferase, U/L | 15.0-40.0 | 27.0 (21.0-35.1) | 33.0 (24.9-43.5) | 25.0 (20.0-33.0) | <0.001 |
| Total bilirubin, μmol/L | 0.0-21.0 | 9.8 (7.8-13.8) | 9.6 (8.3-15.4) | 9.9 (7.6-13.7) | 0.295 |
| Serum creatinine, μmol/L | 57.0-111.0 | 63.0 (51.0-76.0) | 71.1 (53.0-84.5) | 61.0 (51.0-74.4) | 0.006 |
| Blood urea nitrogen, mmol/L | 3.6-9.5 | 3.9 (3.2-4.9) | 4.5 (3.4-5.7) | 3.8 (3.1-4.6) | <0.001 |
| Creatine kinase, U/L | 50.0-310.0 | 61.0 (45.5-95.5) | 68.0 (50.3-131.8) | 59.0 (43.5-89.0) | 0.025 |
| NT-pro BNP, pg/mL | 0-125.0 | 37.2 (24.7-67.3) | 57.0 (36.2-96.0) | 34.4 (23.8-61.3) | <0.001 |
| Lactate dehydrogenase, U/L | 120.0-350.0 | 227.0 (175.0-425.0) | 322.0 (197.5-517.5) | 217.0 (168.0-400.0) | <0.001 |
| Glucose, mmol/L | 3.9-6.1 | 5.8 (5.2-6.9) | 5.9 (5.3-7.8) | 5.7 (5.1-6.6) | 0.015 |
| **Arterial blood gas** | | | | | |
| Sodium, mmol/L | 135.0-145.0 | 138.8 (137.0-140.1) | 138.0 (135.5-139.8) | 138.9 (137.3-140.3) | 0.001 |
| Potassium, mmol/L | 3.5-5.0 | 3.8 (3.6-4.1) | 3.8 (3.5-4.1) | 3.9 (3.6-4.1) | 0.048 |
| Chloride, mmol/L | 90.0-110.0 | 104.0 (101.6-106.0) | 103.0 (100.0-106.0) | 104.0 (102.0-106.0) | 0.012 |
| PaO_2_, mm Hg | 83.0-108.0 | 92.7 (81.9-106.5) | 81.2 (72.0-94.3) | 96.9 (86.8-112.5) | <0.001 |
| PaCO_2_, mm Hg | 35.0-48.0 | 38.9 (36.3-41.1) | 38.7 (34.1-41.0) | 39.0 (37.0-41.2) | 0.045 |
| PaO_2_:FIO_2_, mm Hg | 400.0-500.0 | 423.8 (361.4-477.3) | 339.5 (238.8-384.8) | 443.8 (401.2-488.7) | <0.001 |
| Lactate, mmol/L | 0.5-1.6 | 1.2 (1.0-1.6) | 1.2 (1.0-1.6) | 1.2 (1.0-1.6) | 0.999 |
| **Immune-related biomarkers** | | | | | |
| IgG, g/L | 7.0-16.0 | 11.7 (10.1-13.3) | 10.9 (9.6-13.1) | 11.8 (10.3-13.4) | 0.326 |
| IgA, g/L | 0.7-4.0 | 2.0 (1.5-2.7) | 1.9 (1.4-2.8) | 2.0 (1.5-2.6) | 0.558 |
| IgM, g/L | 0.4-2.5 | 1.0 (0.7-1.2) | 0.7 (0.5-1.1) | 1.0 (0.8-1.3) | 0.001 |
| IgE, IU/mL | 0.0-100.0 | 57.4 (20.8-166.5) | 57.4 (20.1-122.6) | 55.9 (21.1-169.1) | 0.752 |
| C3c, mean (SD), g/L | 0.9-1.8 | 1.1 (0.2) | 1.1 (0.2) | 1.1 (0.2) | 0.692 |
| C4, mean (SD), g/L | 0.1-0.4 | 0.3 (0.1) | 0.3 (0.1) | 0.3 (0.1) | 0.132 |
| Absolute T lymphocyte counts, count/μL | NA | 999.5 (649.0-1400.5) | 687.0 (474.3-1117.0) | 1060.5 (718.0-1434.0) | <0.001 |
| T lymphocyte ratio, % | 65.0-79.0 | 67.4 (59.1-73.0) | 62.9 (50.7-68.4) | 69.0 (61.6-74.2) | <0.001 |
| Absolute helper T lymphocyte counts, count/μL | NA | 542.5 (376.8-759.0) | 431.5 (241.3-653.3) | 590.5 (401.5-782.8) | 0.001 |
| Helper T lymphocyte ratio, % | 34.0-52.0 | 36.8 (31.5-42.6) | 36.1 (25.9-42.8) | 37.1 (32.3-42.2) | 0.081 |
| Absolute cytoxic T lymphocyte counts, count/μL | NA | 344.5 (214.8-523.3) | 243.0 (149.3-416.8) | 373.0 (247.0-562.8) | <0.001 |
| Cytoxic T lymphocyte ratio, % | 21.0-39.0 | 23.7 (18.2-29.2) | 21.6 (16.1-25.3) | 24.9 (18.9-31.0) | 0.003 |
| CD4/CD8 ratio | 0.9-3.6 | 1.6 (1.1-2.1) | 1.7 (1.2-2.2) | 1.5 (1.1-2.1) | 0.369 |
| **Inflammation-related biomarkers** | | | | | |
| C-reactive protein, mg/L | 0.0-10.0 | 11.8 (3.6-26.5) | 24.5 (9.3-53.7) | 8.8 (3.1-22.2) | <0.001 |
| Procalcitonin, ng/mL | 0.0-5.0 | 0.05 (0.03-0.07) | 0.04 (0.03-0.06) | 0.06 (0.04-0.09) | <0.001 |
| Interleukin-6, pg/mL | 0.0-7.0 | 12.7 (4.9-20.7) | 19.6 (13.3-47.8) | 10.1 (3.9-18.5) | <0.001 |
| Erythrocyte sedimentation rate, mm/h | 0.0-15.0 | 28.0 (14.0-45.5) | 40.0 (20.0-57.0) | 25.0 (13.0-41.3) | <0.001 |

Abbreviations: BNP, Brain natriuretic peptide; NA, not applicable; SD, standardized differences. Data were median (IQR) if not otherwise specified. n (%) referred to the total number of patients with available data. P values indicated differences between sepsis and non-sepsis patients, in which *P* < 0.05 was deemed as statistical significance.
